# American Heart Association/American Stroke Association Deletes Sections from 2018 Stroke Guidelines

**DOI:** 10.5811/westjem.2018.9.39659

**Published:** 2018-10-22

**Authors:** C. Eric McCoy, Mark I. Langdorf, Shahram Lotfipour

**Affiliations:** University of California Irvine School of Medicine, Department of Emergency Medicine, Orange, California

## Abstract

The updated American Heart Association (AHA)/American Stroke Association (ASA) Guidelines for the Early Management of Patients with Acute Ischemic Stroke were published in January 2018.^1^ The purpose of the guidelines is to provide an up-to-date, comprehensive set of recommendations for clinicians caring for adult patients with acute arterial ischemic stroke in a single document. The guidelines detail new and updated recommendations that reflect and incorporate the most recent literature in the evaluation and management of acute ischemic stroke. Some sections of the latest guidelines have sparked debate in the medical community.

Debate with regard to deciding the optimal diagnostic and treatment strategy for patients is healthy and anticipated with the release of new medical literature or recommendations. However, what is somewhat puzzling and unanticipated with the release of these new guidelines is that within two months of their release the AHA/ASA rescinded its recently released guidelines, publishing a “correction” in which several parts of the document have been deleted.^2^ An action such as this at the guideline level is unprecedented in recent history and has left stakeholders in the medical community somewhat confused as to the rationale for its occurrence. This article will inform the emergency medicine (EM) healthcare professional of the recent correction of the updated stroke guidelines, identify which sections have been removed (deleted), and will provide a brief summary of the pertinent updates (that have not been deleted) to the 2018 stroke guidelines that have particular relevance to the EM community.

## INTRODUCTION

The American Heart Association (AHA)/American Stroke Association (ASA) has released a correction to its “2018 Guidelines for the Early Management of Patients with Acute Ischemic Stroke: A Guideline for Healthcare Professionals from the American Heart Association/American Stroke Association.”^1^,^2^ In this correction notification, the AHA/ASA reports that based on recent feedback received from the clinical stroke community related to the “2018 Guidelines . . .,” which published ahead of print January 24, 2018, and appeared in the March 2018 issue of the journal, the AHA/ASA has reviewed the guidelines and is preparing clarifications, modifications, and/or updates to several sections. Additionally, several sections were deleted from the guidelines^2^ (Table 1).

Although the correction document reports continued support for the corrected version of the guidelines and its support for clinical decision-making, the rescinding of sections of the guidelines was done without the agreement of the Guideline Writing Committee.^3^ Chair of the Guideline Writing Committee, William J Powers, MD, H. Houston Merritt Distinguished Professor and Chair, Department of Neurology, University of North Carolina at Chapel Hill, told Medscape Medical News: “This action by the AHA was carried out against the strongly voiced opposition and without the agreement of the majority of the 2018 Acute Ischemic Stroke Writing Group.”^3^ He also commented on contentious sections that had been deleted such as brain imaging recommendations and dysphagia recommendations. Powers reported, “in the case of MRI [magnetic resonance imaging] scans (referring to specific types), we simply stated they don’t need to be routinely performed in all patients. There are certain patients in whom you have all the information you need to provide excellent, evidence-based patient care without an MRI scan. We didn’t state that MRI scans should never be done in anyone, just each patient should be considered individually in deciding whether MRI would be of benefit.”^3^ Regarding dysphagia screening, he reported that . . . “we didn’t think there was enough evidence to recommend that every patient must have this. We said it was reasonable, but not mandatory.”^3^ Powers reports that the writing committee is working closely with the AHA to address the issues that have been raised. In a statement sent to Medscape Medical News, the AHA reported that they have reconvened the writing group to consider whether clarifications, modifications, or updates would address the concerns and anticipate an updated version of the guidelines to be ready for publication by summer 2018.^3^

### Selected New Recommendations Pertinent to the Emergency Medicine Provider

Although with several new recommendations, there are a few new updates that bear particular relevance to emergency healthcare providers working in the prehospital and emergency department setting. One of the updates purported to have a significant impact on the initial evaluation and management of patients with suspected acute ischemic stroke is the window of time to perform endovascular thrombectomy, being increased to up to 24 hours in carefully selected patients. These recommendations are based on the results from the DAWN and DEFUSE 3 trials, which evaluated the effectiveness of endovascular therapy (thrombectomy) plus standard care vs. standard care alone in patients with large vessel occlusion acute ischemic stroke who had last been known to be well 6 to 24 hours (24 for DAWN and 16 hours for DEFUSE 3 studies) earlier with specific findings on advanced neuroimaging.^4^,^5^ The primary outcomes that were measured focused on disability (e.g., utility-weighted modified Rankin scale) at 90 days. The authors concluded that among patients with acute stroke last known to be well 6 to 24 hours (24 for DAWN, 16 for DEFUSE 3) earlier who had a mismatch between clinical deficit and infarct, outcomes for disability at 90 days were better with thrombectomy plus standard care than with standard care alone. The authors observed no significant difference in symptomatic intracranial hemorrhage or death.^4^,^5^

The extension of the window of thrombectomy for ischemic stroke patients up to 24 hours is particularly significant as this new extended window can increase the proportion of patients who may benefit from this intervention. Noting that the inclusion criteria is selective and only a subset of the acute ischemic stroke population will benefit, this extended therapeutic window for this intervention has tremendous implications for the prehospital care environment where systems are designed to transport patients to healthcare facilities that can optimize the likelihood of positive patient outcomes. Regionalization of diagnosis-specific care through specialty centers and transporting patients to those centers equipped with the human and capital resources to achieve optimal patient outcomes takes a tremendous amount of time, energy, effort, planning, and resources at the organizational level of any emergency medical services (EMS) system. These changes also have implications for protocols and procedures at the hospital and provider level. This may be one of the reasons why so much consternation over the new recommendation commenting on bypassing intravenous (IV) alteplase-capable hospitals to transport to a higher level of stroke care may have been deleted. Specifically, Section 1.3 EMS Systems, recommendation 4 stated that when several IV alteplase-capable hospital options exist within a defined geographic region, the benefit of bypassing the closest to bring the patient to one that offers a higher level of stroke care, including mechanical thrombectomy, is uncertain.^1^ From the removal of this recommendation, it is clear that different stakeholders have varying opinions with regard to optimal facility for acute ischemic stroke patients in the prehospital setting. Along with direct medical management, the guidelines also have implications for policies, protocols, procedures, financing, and operations at the systems level.

The use of telemedicine evaluation of acute ischemic stroke patients is also a new recommendation that supports a service to assist community physicians who do not have access to on-site neurological services. Telemedicine allows physicians and patients in resource-poor (specifically, neurology resources) communities to benefit from the expertise of a neurology consultation via live audio/video communication or simply by phone. The recommendations report that the administration of IV alteplase guided by telestroke consultation for patients with acute ischemic stroke may be as safe and beneficial as that of stroke centers.^1^ Telemedicine provides the opportunity to extend the benefit of evidence-based decision-making to areas lacking the appropriate human resources. Other notable new updates include a secondary goal of door-to-needle time of 45 minutes in more than 50% of patients (primary goal stands at 60 minutes), performing brain imaging with 20 minutes of patient arrival in more than 50% of patients, and the use of brief, moderate hyperventilation (PCO_2_ target 30–34 millimeters of mercury) for patients with acute severe neurological decline from brain swelling as a bridge to more definitive therapy.^1^ For a selected list of new recommendations from the 2018 AHA/ASA Stroke Guidelines pertinent to the emergency practitioner in the acute initial setting, see table Table 2.

## SUMMARY

The AHA/ASA has released a correction to the 2018 Guidelines for the Early Management of Patients with Acute Ischemic Stroke: A Guideline for Healthcare Professionals from the American Heart Association/American Stroke Association since its initial online publication in January 2018.^1^,^2^ In this correction notification, the AHA/ASA reported that based on recent feedback received from the clinical stroke community related to the “2018 Guidelines” . . . the AHA/ASA has reviewed the guidelines and is preparing clarifications, modifications, and/or updates to several sections in it. Several sections of guideline were removed and the rescinding of the guidelines was done without the agreement of the Guideline Writing Committee.^3^ Among those sections removed were ones that caused substantive debate within the medical community and relevant stakeholders. However, the updated 2018 Guidelines provide the latest treatment recommendations for patients with acute ischemic stroke and within the standing sections of the corrected version, provide the emergency provider with the latest evidence-based updates.

## Figures and Tables

**Table 1:** Sections that were deleted from the 2018 Stroke Guidelines.

Section	Content deleted from guideline
Section 1.3	EMS systems recommendation 4
Section 1.4	Hospital stroke capabilities recommendation 1
Section 1.6	Telemedicine recommendation 3
Section 2.2	Brain imaging recommendation 11
Section 3.2	Blood pressure recommendation 3
Section 4.3	Blood pressure recommendation 2
Section 4.6	Dysphagia recommendation 1
Section 6.0	All subsections (11)

*EMS,* emergency medical services.

**Table 2:** Selected new recommendations from 2018 AHA/ASA Stroke Guidelines pertinent to the emergency practitioner.

Section	Pertinent content for the emergency provider in the acute initial setting
Section 1.5 Hospital stroke teams	1.5.3 It may be reasonable to establish a secondary DTN time goal of within 45 minutes in > 50% of patients with AIS treated with IV alteplase.
	1.5.5 Multicomponent quality improvement initiatives, which include ED education and multidisciplinary teams with access to neurological expertise, are recommended to safely increase IV thrombolytic treatment.
Section 1.6 Telemedicine	1.6.4 Telestroke/teleradiology evaluations of AIS patients can be effective for correct IV alteplase eligibility decision making.
	1.6.5 Administration of IV alteplase guided by telestroke consultation for patients with AIS may be as safe and as beneficial as that of stroke centers.
	1.6.6 Providing alteplase decision-making support via telephone to community physicians is feasible and safe and may be considered when a hospital has access to neither an in-person stroke team nor a telestroke system.
	1.6.7 Telestroke networks may be reasonable for triaging patients with AIS who may be eligible for interfacility transfer in order to be considered for acute mechanical thrombectomy.
Section 2.2 Brain imaging	2.2.2 Systems should be established so that brain imaging studies can be performed within 20 minutes of arrival in the ED in at least 50% of patients who may be candidates for IV alteplase and/or mechanical thrombectomy.
	2.2.4 The CT hyperdense MCA sign should not be used as a criterion to withhold IV alteplase from patients who otherwise qualify.
	2.2.5 Routine use of magnetic resonance imaging (MRI) to exclude cerebral microbleeds (CMBs) before administration of IV alteplase is not recommended.
	2.2.7 Multimodal CT and MRI, including perfusion imaging, should not delay administration of alteplase.
	2.2.9 For patients who otherwise meet criteria for EVT, it is reasonable to proceed with CTA if indicated in patients with suspected intracranial LVO before obtaining a serum creatinine concentration in patients without a history of renal impairment.
	2.2.10 In patients who are potential candidates for mechanical thrombectomy, imaging of the extracranial carotid and vertebral arteries, in addition to the intracranial circulation, is reasonable to provide useful information on patient eligibility and endovascular procedural planning.
	2.2.12 In selected patients with AIS within 6 to 24 hours of last known normal who have LVO in the anterior circulation, obtaining CTP, DW-MRI, or MRI perfusion is recommended to aid in the patient selection for mechanical thrombectomy, but only when imaging and other eligibility criteria from RCTs showing benefit are being strictly applied in selecting patients for mechanical thrombectomy.
Section 3.2 Blood pressure	3.2.1 Hypotension and hypovolemia should be corrected to maintain systemic perfusion levels necessary to support organ function.
Section 3.5 IV Alteplase	3.5.3 For otherwise eligible patients with mild stroke presenting in the 3- to 4.5-hour window, treatment with IV alteplase may be reasonable.
	3.5.4 In otherwise eligible patients who have had a previously demonstrated small number (1–10) of CMBs on MRI, administration if IV alteplase is reasonable.
	3.5.5 In otherwise eligible patients who have a previously demonstrated high burden of CMBs (>10) on MRI, treatment with IV alteplase may be associated with an increase risk of sICH, and the benefits of treatment are uncertain.
	3.5.6 IV alteplase for adults presenting with an AIS with known sickle cell disease can be beneficial.
	3.5.15 The risk of antithrombotic therapy within the first 24 hours after treatment with IV alteplase (with or without EVT) is uncertain.
3.6 Other IV Thrombolytics and sonothrombolytics	3.6.2 Tenecteplase administered as a 0.4 mg/kg single IV bolus has not been proven to be superior or noninferior to alteplase but might be considered as an alternative to alteplase in patients with minor neurological impairment and no major intracranial occlusion.
3.7 Mechanical thrombectomy	3.7.7 In selected patients with AIS within 6 to 16 hours of last known normal who have LVO in the anterior circulation and meet other DAWN or DEFUSE 3 eligibility criteria, mechanical thrombectomy is recommended.
	3.7.8 In selected patients with AIS within 6 to 24 hours of last known normal who have LVO in the anterior circulation and meet other DAWN eligibility criteria, mechanical thrombectomy is reasonable.
	3.7.17 In patients who undergo mechanical thrombectomy, it is reasonable to maintain BP < 180/105 mm Hg during and for 24 hours after the procedure.
	3.7.18 In patients who undergo mechanical thrombectomy with successful reperfusion, it might be reasonable to maintain BP at a level < 180/105 mmHg.
3.9 Antiplatelet treatment	3.9.5 In patients with minor stroke, treatment for 21 days with dual antiplatelet therapy (aspirin and clopidogrel) begun within 24 hours can be beneficial for early secondary stroke prevention for a period of up to 90 days from symptom onset.
3.10 Anticoagulants	3.10.3 The safety and usefulness of short-term anticoagulation for nonocclusive, extracranial intraluminal thrombus in the setting of AIS are not well established.
	3.10.5 The safety and usefulness of factor Xa inhibitors in the treatment of AIS are not well established.
4.3 Blood pressure	4.3.1 In patients with AIS, early treatment of hypertension is indicated when required by comorbid conditions. Lowering BP initially by 15% is probably safe.
	4.3.3 In patients with BP > 220/120 mmHg who do not receive IV alteplase or EVT and have no comorbid conditions requiring acute antihypertensive treatment, the benefit of initiating or reinitiating treatment of hypertension within the first 48 to 72 hours is uncertain. It might be reasonable to lower BP by 15% during the first 24 hours after onset of stroke.
	4.3.5 Starting or restarting antihypertensive therapy during hospitalization in patients with BP >140/90 mmHg who are neurologically stable is safe and is reasonable to improve long-term BP control unless contraindicated.
	4.3.6 Hypotension and hypovolemia should be corrected to maintain systemic perfusion levels necessary to support organ function.
4.8 Deep vein thrombosis Prophylaxis	4.8.2 The benefit of prophylactic-dose subcutaneous heparin (unfractionated heparin [UFH] or LMWH) in immobile patients with AIS is not well established.
	4.8.3 When prophylactic anticoagulation is used, the benefit of prophylactic-dose LMWH over prophylactic-dose UFH is uncertain.
5.1 Cerebellar and cerebral edema	5.1.4 Patients with large territorial supratentorial infarctions are at high risk for complicating brain edema and increased intracranial pressure. Discussion of care options and possible outcomes should take place quickly with patients (if possible) and caregivers. Medical professionals and caregivers should ascertain and include patient-centered preferences in shared decision making, especially during prognosis formation and considering interventions or limitations of care.
	5.1.10 Use of brief moderate hyperventilation (PCO2 target 30–34 mmHg) is a reasonable treatment for patients with acute severe neurological decline from brain swelling as a bridge to more definitive therapy.

*AHA,* American Heart Association*; ASA,* American Stroke Association*; DTN,* door-to-needle; *AIS,* acute ischemic stroke; *BP,* blood pressure; *CMB,* cerebral microbleed; *CT,* computed tomography; *MCA,* middle cerebral artery; CTA, computed tomography angiography; *DW-MRI,* diffusion weighted magnetic resonance imaging; *ED,* emergency department; *IV,* intravenous; *LVO,* large vessel occlusion; *MRI,* magnetic resonance imaging; *sICH,* symptomatic intracerebral hemorrhage.

*DTN,* door-to-needle; *AIS,* acute ischemic stroke; *BP,* blood pressure; *CMB,* cerebral microbleed; *CT,* computed tomography; *DW-MRI,* diffusion weighted magnetic resonance imaging; *ED,* emergency department; *IV,* intravenous; *LVO,* large vessel occlusion; *MRI,* magnetic resonance imaging; *sICH,* symptomatic intracerebral hemorrhage; PCO2; partial pressure of carbon dioxide.

(Note: This list provides only a selected list of new recommendations introduced in the guidelines and is not exhaustive; for further details refer to the comprehensive guideline document.^1^)
